# Treatment-free remission following frontline nilotinib in patients with chronic myeloid leukemia in chronic phase: results from the ENESTfreedom study

**DOI:** 10.1038/leu.2017.63

**Published:** 2017-03-17

**Authors:** A Hochhaus, T Masszi, F J Giles, J P Radich, D M Ross, M T Gómez Casares, A Hellmann, J Stentoft, E Conneally, V García-Gutiérrez, N Gattermann, W Wiktor-Jedrzejczak, P D le Coutre, B Martino, S Saussele, H D Menssen, W Deng, N Krunic, V Bedoucha, G Saglio

**Affiliations:** 1 Abteilung Hämatologie/Onkologie, Universitätsklinikum Jena, Jena, Germany; 2 Department of Haematology and Stem Cell Transplantation St István and St László Hospital, Budapest, Hungary; 3 Developmental Therapeutics Program, Feinberg School of Medicine, Northwestern University, Chicago, IL, USA; 4 Clinical Research Division, Fred Hutchinson Cancer Research Center, Seattle, WA, USA; 5 SA Pathology, Adelaide, SA, Australia; 6 Hospital Universitario Doctor Negrin, Las Palmas de Gran Canaria, Spain; 7 Medical University of Gdańsk, Gdańsk, Poland; 8 Aarhus University Hospital, Aarhus, Denmark; 9 St James Hospital, Dublin, Ireland; 10 Servicio de Hematología, Hospital Universitario Ramón y Cajal (IRYCIS), Madrid, Spain; 11 Universitätsklinikum Düsseldorf, Düsseldorf, Germany; 12 Medical University of Warsaw, Department of Hematology, Warsaw, Poland; 13 Charité Universitätsmedizin Berlin, Berlin, Germany; 14 Azienda Ospedaliera Bianchi Melacrino Morelli, Reggio Calabria, Italy; 15 III. Med. Klinik, Medizinische Fakultät Mannheim der Universität Heidelberg, Mannheim, Germany; 16 Novartis Pharma AG, Basel, Switzerland; 17 Novartis Pharmaceuticals Corporation, East Hanover, NJ, USA; 18 Novartis Institute for Biomedical Research, Cambridge, MA, USA; 19 University of Turin, Orbassano, Italy

## Abstract

The single-arm, phase 2 ENESTfreedom trial assessed the potential for treatment-free remission (TFR; i.e., the ability to maintain a molecular response after stopping therapy) following frontline nilotinib treatment. Patients with Philadelphia chromosome-positive chronic myeloid leukemia in chronic phase with MR^4.5^ (*BCR-ABL1*⩽0.0032% on the International Scale (*BCR-ABL1*^IS^)) and ⩾2 years of frontline nilotinib therapy were enrolled. Patients with sustained deep molecular response during the 1-year nilotinib consolidation phase were eligible to stop treatment and enter the TFR phase. Patients with loss of major molecular response (MMR; *BCR-ABL1*^IS^⩽0.1%) during the TFR phase reinitiated nilotinib. In total, 215 patients entered the consolidation phase, of whom 190 entered the TFR phase. The median duration of nilotinib before stopping treatment was 43.5 months. At 48 weeks after stopping nilotinib, 98 patients (51.6% 95% confidence interval, 44.2–58.9%) remained in MMR or better (primary end point). Of the 86 patients who restarted nilotinib in the treatment reinitiation phase after loss of MMR, 98.8% and 88.4%, respectively, regained MMR and MR^4.5^ by the data cutoff date. Consistent with prior reports of imatinib-treated patients, musculoskeletal pain-related events were reported in 24.7% of patients in the TFR phase (consolidation phase, 16.3%).

## Introduction

Treatment-free remission (TFR; that is, sustained major molecular response (MMR; *BCR-ABL1*⩽0.1% on the International Scale (*BCR-ABL1*^IS^)) or deep molecular response (DMR) after stopping tyrosine kinase inhibitor (TKI) therapy) is an emerging treatment goal for patients with chronic myeloid leukemia in chronic phase (CML-CP). Current recommendations of the European LeukemiaNet call for indefinite treatment with TKIs in responding patients,^1^ whereas the 2017 National Comprehensive Cancer Network guidelines for CML note that discontinuation of TKI therapy is feasible in selected patients in the context of careful monitoring.^2^ Patients may be motivated to stop therapy for reasons including relief from treatment side effects, the ability to safely attempt pregnancy or the desire for other potential improvements in health-related quality of life.^3, 4, 5^ The potential for patients who achieve DMR to remain in remission after stopping TKI therapy was first demonstrated in the Stop Imatinib 1 trial, in which ≈40% of patients with sustained DMR (with undetectable *BCR-ABL1* for ⩾2 years) on long-term imatinib maintained this response (no confirmed loss of undetectable *BCR-ABL1*) 1 year after stopping treatment.^6^

Since the Stop Imatinib 1 trial, several additional trials have investigated TKI discontinuation in patients with sustained DMR, the majority of which involved patients treated with long-term imatinib therapy.^4, 7, 8, 9, 10, 11, 12, 13^ However, the European Stop Tyrosine Kinase Inhibitor (EURO-SKI) study—the largest TFR trial to date^10^—includes patients who achieved sustained DMR (specifically, MR^4^ (*BCR-ABL1*^IS^⩽0.01%) for ⩾1 year) on imatinib, nilotinib or dasatinib. Other ongoing trials are specifically investigating TFR following treatment with second-generation TKIs, including the STOP 2G-TKI study of patients with undetectable *BCR-ABL1* for ⩾2 years on frontline or second-line nilotinib or dasatinib,^14^ the Dasatinib Discontinuation study of patients on second- or later-line dasatinib with DMR (*BCR-ABL1*<0.0069%) for ⩾1 year^12^ and a series of four Evaluating Nilotinib Efficacy and Safety in Clinical Trials (ENEST) studies assessing TFR in nilotinib-treated patients: ENESTfreedom in patients administered frontline nilotinib and ENESTop, ENESTgoal and ENESTpath in distinct populations of patients administered second-line nilotinib.^15, 16, 17^ The ongoing single-arm, phase 2 ENESTfreedom trial is the first study to assess specifically the potential for patients with sustained DMR during frontline nilotinib to stop treatment. Here we present the first results and the results of the primary end point analysis from ENESTfreedom with a minimum follow-up of 48 weeks after stopping nilotinib.

## Materials and methods

### Patients, study design and treatment

Patients (aged ⩾18 years) with Philadelphia chromosome-positive CML-CP who had ⩾2 years of frontline treatment with nilotinib and achieved MR^4.5^ (*BCR-ABL1*^IS^⩽0.0032% determined by a designated laboratory (MolecularMD, Portland, OR, USA) in a sample with ⩾32 000 *ABL1* copies) were eligible. Evidence of typical *BCR-ABL1* transcripts (that is, b3a2 (e14a2) and/or b2a2 (e13a2)) at the time of diagnosis was required. Patients who had previously received other BCR-ABL1 TKIs (cumulative duration >4 weeks) or interferon-alfa (any duration) or were unable to tolerate a minimum dose of nilotinib 400 mg once daily were not eligible.

**Figure 1:**
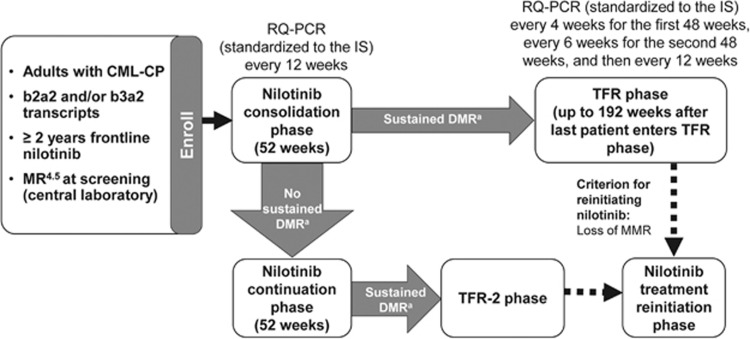
ENESTfreedom study design.^a^Defined as (in the last 4 quarterly RQ-PCR assessments) MR^4.5^ in the last assessment, no assessment worse than MR^4^ and ⩽2 assessments between MR^4^ and MR^4.5^.

Upon enrollment all patients entered a 1-year consolidation phase during which they continued nilotinib treatment (Figure 1). Molecular responses were assessed every 12 weeks during the year of consolidation treatment by real-time quantitative polymerase chain reaction (RQ-PCR); sustained DMR was defined as MR^4.5^ in the last assessment, no assessment worse than MR^4^ and⩽2 assessments between MR^4^ and MR^4.5^. Patients with sustained DMR throughout the consolidation phase were eligible to enter the TFR phase and stop nilotinib treatment. Patients with loss of MMR during the TFR phase were required to reinitiate nilotinib 300 mg two times daily (or their previously tolerated dose) in the treatment reinitiation phase. Patients who did not sustain DMR during the consolidation phase remained on nilotinib treatment (continuation phase; Supplementary Appendix).

### Study end points and assessments

The primary efficacy end point was the proportion of patients who were in MMR without reinitiation of treatment at week 48 of the TFR phase. Secondary end points included the proportion of patients who were in MR^4.5^ and off treatment at 48 weeks from the start of the TFR phase, treatment-free survival (TFS) over time (defined as the time from the start of TFR until the earliest occurrence of any of the following: loss of MMR, reinitiation of nilotinib for any reason, progression to accelerated phase (AP)/blast crisis (BC) or death because of any cause), reachievement of MMR and MR^4.5^ after nilotinib reinitiation and safety.

Molecular responses were measured as ratios of *BCR-ABL1* to *ABL1* expressed on the IS, determined from RQ-PCR assessments of peripheral blood conducted in a central laboratory. During the TFR phase, molecular responses were evaluated every 4 weeks for the first 48 weeks, every 6 weeks for the next 48 weeks and every 12 weeks thereafter. Patients with loss of MR^4^ during the TFR phase were monitored every 2 weeks until they regained MR^4^, remained in MMR without regaining MR^4^ for 8 weeks (at which point the patient received monthly monitoring) or lost MMR and entered the treatment reinitiation phase. During the treatment reinitiation phase, molecular responses were evaluated every 4 weeks for the first 24 weeks and every 12 weeks thereafter (or as clinically indicated for patients who had not regained MMR). When sufficient sample was available, mutational analysis by Sanger sequencing was performed for patients with loss of MMR, or as clinically indicated.

Adverse events (AEs) were assessed according to the Common Terminology Criteria for Adverse Events version 4.03. Evaluation of AEs and laboratory abnormalities were conducted on an ongoing basis on study and for up to 30 days after the last dose of study treatment or last day of TFR.

The impact of TFR on patients’ quality of life was assessed as an exploratory end point. At protocol-defined time points throughout the study, patient-reported outcomes were collected using the MD Anderson Symptom Inventory for CML and EuroQol visual analog scale to obtain overall quality-of-life scores, as well as the EuroQol EQ-5D-5L questionnaire, in which patients reported the presence or absence and severity of problems experienced related to mobility, self-care, usual activities, pain/discomfort and anxiety/depression.^18, 19^

### Statistical analyses

The planned sample size was based on Fleming’s single-stage design and the minimum number of patients required to reject the null hypothesis for the primary end point (⩽50% TFR rate at 48 weeks). Assuming that ≈30% of enrolled patients would not qualify for the TFR phase, a minimum enrollment of 175 patients was required to achieve 90% power to reject the null hypothesis with a one-sided *α*-level of 2.5% if the true TFR rate at 48 weeks was ⩾65%. With an actual enrollment of 215 patients, the power increased to 94% with all the other assumptions being the same.

Demographics, baseline characteristics, efficacy and safety results are reported for patients who entered the TFR phase (that is, the TFR population). The primary end point was presented as a percentage with an exact 95% Clopper–Pearson confidence interval (CI). TFS was estimated using the Kaplan–Meier method; survival time for patients without an event was censored at the date of the last assessment. Rates of MMR and MR^4.5^ regained in the treatment reinitiation phase were reported as cumulative incidences. An analysis of baseline factors as potential predictors of TFR maintenance was conducted using a multivariate logistic regression analysis. The MMR rate at 48 weeks and TFS were also analyzed in patients who stayed in the TFR phase for ⩾24 weeks. The frequencies of AEs, laboratory abnormalities and predefined groupings of AE types of special interest were summarized for the TFR population by study phase (the consolidation phase and the first 48 weeks of the TFR phase).

The data presented herein are based on a cutoff date of 30 November 2015, at which time all patients who entered the TFR phase had completed ⩾48 weeks of TFR, entered the treatment reinitiation phase or discontinued the study.

### Ethics

This study was designed and conducted in accordance with the ICH Harmonized Tripartite Guidelines for Good Clinical Practice and the ethical principles of the Declaration of Helsinki. All patients provided written informed consent before any study procedures and in accordance with local laws/regulations. The study protocol and amendments were reviewed by an independent ethics committee or institutional review board for each study center.

## Results

### Patients

**Figure 2:**
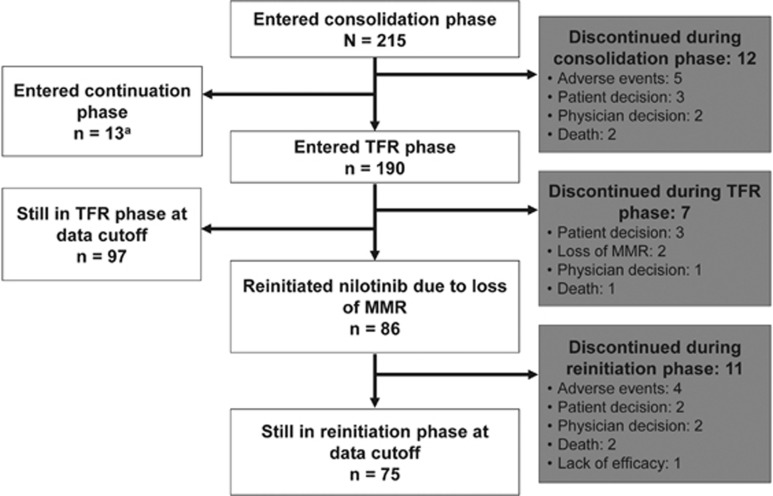
Patient disposition. ^a^Three of these patients were still in the continuation phase at the data cutoff date. Of the 10 patients who completed the continuation phase, 8 were in the TFR-2 phase and 2 remained on nilotinib therapy at the data cutoff date.

A total of 231 patients were screened for eligibility between 4 March 2013 and 22 November 2013; 215 of these patients enrolled and entered the consolidation phase. Two hundred and three patients completed the consolidation phase, including 190 (88.4%) who entered the TFR phase and 13 who entered the continuation phase (due to no sustained DMR during the consolidation phase). Twelve patients discontinued from the study during the consolidation phase (Figure 2), including two patients who discontinued with sustained DMR (due to patient decision). In the TFR population (*n*=190), the median time from first achievement of MR^4.5^ to study entry was 18.3 months, and the median duration of nilotinib before TFR was 43.5 months (Table 1).

### TFR phase

**Figure 3:**
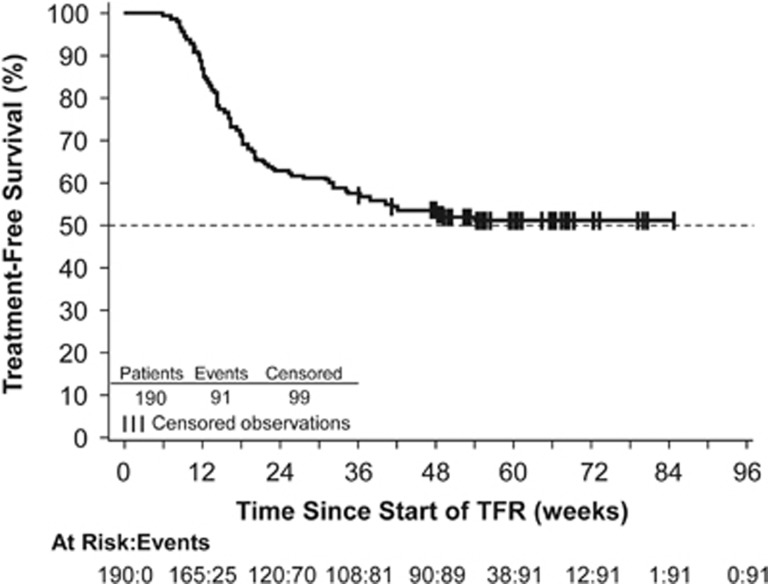
Kaplan–Meier estimate of TFS among all patients who entered the TFR phase. TFS was defined as the time from the start of TFR until the earliest of any of the following: loss of MMR, reinitiation of nilotinib for any reason, progression to AP/BC or death because of any cause.

At week 48 of the TFR phase, 98 patients (51.6% 95% CI, 44.2–58.9 (null hypothesis could not be rejected because of the lower limit of the 95% CI being ⩽50%)) remained in MMR without treatment reinitiation; of these 98 patients, 5 had confirmed loss of MR^4^ by 48 weeks (1 of these 5 patients went on to lose MMR after 48 weeks but before the data cutoff date), 3 had loss of MR^4.5^ without confirmed loss of MR^4^ and 90 (47.4% of all 190 patients who stopped treatment; 95% CI, 40.1–54.7) had MR^4.5^ or better at 48 weeks. Ninety-nine of 190 patients did not have a TFS event by the data cutoff date (96 remained in TFR and 3 discontinued from the study (due to patient decision) while in TFR without experiencing a TFS event and were censored in the analysis), whereas 91 patients (47.9%) had TFS events (86 entered the reinitiation phase, 1 died, 3 discontinued TFR without entering the reinitiation phase (1 due to physician decision and 2 due to loss of MMR) and 1 lost MMR at TFR week 48 but remained in the TFR phase at the data cutoff). Most TFS events (70 of 91) occurred within the first 24 weeks (Figure 3); of 120 patients remaining in TFR at 24 weeks, 98 (81.7%) had MMR at 48 weeks (Supplementary Figure S1).

Of the 89 patients with loss of MMR while off nilotinib, 72 had an evaluable mutational assessment, and 1 patient (0.5%) had a detectable *BCR-ABL1* mutation (F359V). Whether this mutation was pre-existing could not be determined because of the low *BCR-ABL1* copy number in all prior samples collected during the study; however, the mutation was not detected in samples collected at initiation of nilotinib therapy and after 2 years on therapy. This patient regained MMR with nilotinib retreatment, subsequently lost MMR on nilotinib, and discontinued from the study owing to the lack of efficacy.

Median age, sex distribution and median durations of nilotinib and DMR were similar among patients with or without TFR at 48 weeks (Supplementary Tables S1 and S2). In a multivariate logistic regression analysis for TFR at 48 weeks with baseline factors as explanatory variables in the model, no strong predictors were formally identified.

### Treatment reinitiation phase

**Figure 4:**
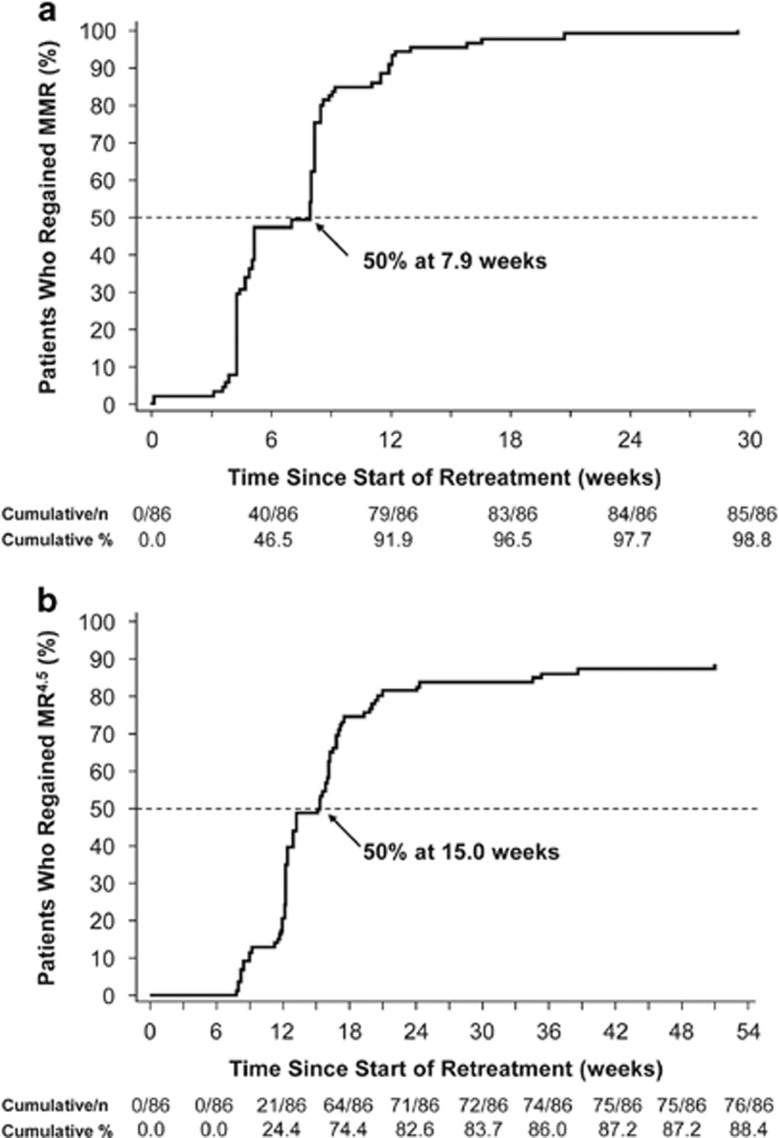
Cumulative incidence of (**a**) MMR and (**b**) MR^4.5^ regained after nilotinib reinitiation.

Of the 86 patients who entered the treatment reinitiation phase (median duration of nilotinib retreatment, 39.6 weeks (range, 5.0–69.7 weeks)), 85 and 76 regained MMR and MR^4.5^, respectively, by the data cutoff date (Figure 4). The patient who did not regain MMR after restarting nilotinib withdrew consent and discontinued from the study after 7.1 weeks of nilotinib retreatment. Of the nine patients who regained MMR but not MR^4.5^ by the data cutoff date, four remained in the treatment reinitiation phase and five discontinued from the study due to AEs (*n*=2), lack of efficacy (*n*=1 (patient with F359V mutation)), physician decision (*n*=1) or patient decision (*n*=1).

### Quality of life

Mean baseline quality-of-life scores were high (mean (s.d.) score at week 48 of the consolidation phase, which was considered the baseline for the TFR phase: MD Anderson Symptom Inventory for CML severity, 1.4 (1.41); MD Anderson Symptom Inventory for CML interference, 1.7 (2.31); EuroQol visual analog scale, 80.5 (15.61)). Minimal changes in quality-of-life scores after stopping or reinitiating treatment were detected among evaluable patients. The proportions of patients reporting problems in each dimension of the EQ-5D-5L tended to be similar across study phases, although a slightly higher proportion of patients reported problems (of any severity) with pain/discomfort after stopping treatment (49.0% of evaluable patients reported problems at week 48 of the consolidation phase compared with 58.1% at week 12 of the TFR phase).

### Safety

No patients progressed to AP/BC. Five deaths were reported by the data cutoff date: two deaths occurred during the consolidation phase (one cardiac arrest and one suicide), one occurred during the TFR phase (unknown cause) and two occurred during the treatment reinitiation phase (one acute myocardial infarction and one due to an unknown cause).

Among patients who entered the TFR phase, AEs were reported in 83.2% during the 1-year consolidation phase (grade 3/4 in 13.7%) and in 65.8% during the first 48 weeks of the TFR phase (grade 3/4 in 11.1%). The most common AEs during the consolidation and TFR phases were nasopharyngitis (11.1%) and arthralgia (12.1%), respectively (Table 2). Elevations in glucose, alanine aminotransferase, aspartate aminotransferase, bilirubin and lipase were less common in the TFR phase than in the consolidation phase. Most patients with laboratory abnormalities during the TFR phase had experienced the same abnormalities during the consolidation phase. No notable differences in hematologic abnormalities were reported during the consolidation phase compared with the TFR phase (Table 2). Cardiovascular events (CVEs) were reported in four patients (2.1%) during the consolidation phase (ischemic heart disease (*n*=2), ischemic cerebrovascular event and peripheral artery disease (*n*=1 each)) and in five patients (2.6%) during the TFR phase (ischemic cerebrovascular events and peripheral artery disease (*n*=2 each) and arteriosclerosis (*n*=1), all of which were first reported during the first 24 weeks of the TFR phase; Table 3). One patient had CVEs reported during both the consolidation and TFR phases. No patient in the TFR population discontinued from the study because of a CVE.

Forty-seven patients (24.7%) had AEs in a predefined musculoskeletal pain grouping (consisting of AEs reported as musculoskeletal pain, myalgia, pain in extremity, arthralgia, bone pain and spinal pain) during the first 48 weeks of the TFR phase compared with 31 patients (16.3%) during the 1-year consolidation phase. Of the 47 patients with musculoskeletal pain-related events in the TFR phase, 45 had grade 1/2 events and 2 had grade 3 events (none led to study discontinuation; including 1 patient with grade 3 arthralgia and 1 patient with 2 episodes of grade 3 bone pain); musculoskeletal pain-related events were more frequent in female (33.0%) than in male patients (18.8%) during the TFR phase (Table 4). Thirty-two of the 47 patients had no history of musculoskeletal pain in the consolidation phase or before enrollment. Most AEs in the musculoskeletal pain grouping (*n*=39) were reported within 24 weeks of stopping nilotinib. The Kaplan–Meier-estimated median duration of these AEs was 24.0 weeks (95% CI, 10.1 weeks—not estimable).

## Discussion

In ENESTfreedom, 51.6% (95% CI, 44.2–58.9) of patients remained in remission at 48 weeks after stopping nilotinib, consistent with results from other TFR studies following long-term TKI treatment.^4, 7, 8, 9, 10, 11, 12, 13^ As the prespecified statistical null hypothesis (TFR rate⩽50%) could not be rejected because of the lower limit of the 95% CI being ⩽50%, the primary end point failed statistically. This is a result of selecting too high of a threshold (50%) for the TFR rate in the null hypothesis, which was due to an underestimation of the impact of the TKI treatment duration on TFR rate at the time the protocol was developed. Nonetheless, the observed TFR rate of 51.6% is a clinically important outcome, particularly when considering the relatively short duration of prior nilotinib therapy (3.6 years) among patients in the study and the association between duration of TKI therapy and TFR probability in prior studies.^4, 10^ Comparison of results across TFR trials is limited by study design variations (for example, depth/duration of DMR required before stopping treatment, minimal duration of TKI therapy before stopping treatment and definition of loss of remission); however, the ability to remain in remission after stopping TKI therapy has been consistently demonstrated in patients with sustained DMR on long-term TKI treatment.^4, 6, 7, 8, 9, 10, 11, 12, 13^ In EURO-SKI, among 772 patients eligible to stop imatinib, nilotinib or dasatinib after a median treatment duration of 91 months and with MR^4^ sustained for ⩾1 year, molecular relapse-free survival at 12 months was 56% (95% CI, 52–59).^10^

Stopping frontline nilotinib was safe in ENESTfreedom: no new safety signals were identified during treatment, nearly all patients who reinitiated nilotinib after loss of MMR regained MMR (98.8%) and MR^4.5^ (88.4%) rapidly, and no patient progressed to AP/BC. The majority of patients with loss of MMR lost the response within 6 months of stopping nilotinib, highlighting the importance of frequent monitoring of patients who stop TKI therapy to ensure timely retreatment. One patient with loss of MMR during the TFR phase was found to have a detectable mutation; however, without serial testing of prior samples, the significance of this finding is unclear because the mutation may have been present before the patient started the TFR phase. Additionally, because low-level mutations can be undetectable using Sanger sequencing,^20^ it is possible that other patients may have had kinase domain mutations below the limit of detection. However, the emergence of *BCR-ABL1* mutations may be less likely during the TFR phase because of the absence of TKI-induced selective pressure for mutants. Kinase domain mutations have not been reported in other TFR studies.^4, 6, 7, 8, 9, 10, 11, 12, 13^

Although long-term safety/tolerability considerations are motivators for stopping treatment in some patients,^3, 4, 5^ the rates of some AEs (for example, hematologic abnormalities and CVEs) were similar in the first 48 weeks of the TFR phase compared with the consolidation phase. This may be due to the fact that this study enrolled patients who had already received nilotinib for multiple years and had established tolerance of nilotinib. Further follow-up is required to determine whether the risk of CVEs and other AEs decreases over time after stopping treatment. In addition, the frequency of low-grade musculoskeletal pain-related events increased during the first year of the TFR phase, consistent with prior reports in patients who stopped imatinib therapy.^4, 21, 22, 23, 24^ In subanalyses of (mostly imatinib-treated) patients from EURO-SKI, ~1/3 experienced musculoskeletal pain after stopping treatment.^21, 22, 24^ Similar events were retrospectively described in 30% (*n*=27/90) of patients in the Korean Imatinib Discontinuation study.^4^ Similar to the findings in ENESTfreedom, the majority of these events were reported within 6 months of stopping therapy.^4^ Many of these events were self-limiting, resolving within 12 months, although some events continued beyond 1 year.^4, 21^

No impact of stopping treatment on patients’ overall quality of life was detected. This may be due to patients having a relatively high quality of life before treatment discontinuation; it may also result from the use of questionnaires that were not optimal for assessment of patients doing well on treatment.

Recent reviews by Saussele *et al.*^25^ and Hughes and Ross^26^ have discussed potential criteria for identifying patients who may be candidates for TFR and recommendations for monitoring of patients during TFR based on available data from clinical trials to date. Both reviews highlight the importance of sustained DMR before stopping treatment and of frequent patient monitoring, including quantification of typical *BCR-ABL1* transcript levels by a well-validated assay run in an IS-standardized center following established guidelines for measuring DMR.^27^ Identification of additional prognostic criteria for TFR remains under investigation.^25, 26^ Although no strong prognostic factors were detected in this study, the analysis may have been limited by the size and relative homogeneity of the population (for example, most patients had a similar prior treatment duration).

Frontline nilotinib therapy is known to result in rapid and high rates of DMR^28, 29^ —a key prerequisite for TFR.^4, 6, 7, 8, 9, 10, 11, 12, 13^ In ENEST1st, 55.2% (*n*=581/1052) and 38.6% (*n*=406/1052) of patients achieved MR^4^ and MR^4.5^, respectively, by 2 years with frontline nilotinib.^29^ In ENESTnd, more than half of patients treated with frontline nilotinib 300 mg two times daily achieved MR^4.5^ by 5 years (*n*=151/282; 54%), compared with 31% of patients treated with frontline imatinib (*n*=89/283).^28^ Furthermore, with 6 years of follow-up in ENESTnd, 38% (*n*=107/282) of patients in the nilotinib 300-mg twice-daily arm vs 22% (*n*=61/283) in the imatinib arm met the treatment duration and sustained DMR requirements defining eligibility to attempt TFR in ENESTfreedom.^30^ These data suggest that frontline nilotinib may increase the number of patients who become eligible to discontinue treatment.

When selecting a TKI for frontline therapy, several factors must be considered: in addition to the efficacy and safety profiles of each available treatment option, treatment cost can be an important consideration, particularly with the introduction of generic imatinib; now, the increased potential for TFR eligibility with nilotinib (and the potential cost savings through treatment discontinuation^31, 32, 33^) may be additional factors to consider for some patients when selecting a frontline TKI. These long-term considerations are increasingly important as patients with CML now have a life expectancy comparable to that of the general population.^34^

As the first study to specifically investigate TFR following frontline nilotinib, ENESTfreedom can provide important new information on this emerging treatment goal for patients with CML-CP. Here, the first results from ENESTfreedom demonstrate that a clinically significant percentage of patients (51.6%) with sustained DMR on frontline nilotinib therapy and a median treatment duration of 43.5 months were able to remain in MMR for ⩾48 weeks after stopping nilotinib. The results from ENESTfreedom, together with the results from ENESTnd showing higher rates of DMR and sustained DMR with nilotinib vs imatinib,^28, 30^ suggest that more patients may become eligible to stop treatment and sustain remission following frontline nilotinib therapy than following imatinib therapy. Additional follow-up and analyses of TFR data in ENESTfreedom and other TFR studies will be needed to further evaluate the patient, disease and treatment characteristics before stopping treatment that may be associated with maintaining TFR, as well as the long-term durability of TFR.

## Figures and Tables

**Table 1:** Baseline characteristics at study entry and nilotinib therapy before TFR phase entry

	*TFR population (*n*=190)*
*Baseline characteristics at study entry*	
Age, median (range) (years)	55.0 (21–86)
Male, *n* (%)	96 (50.5)
Time from CML diagnosis to study entry, median (range) (months)	32.2 (21.4–80.7)
Time from achievement of MR^4.5^ with nilotinib to study entry, median (range) (months)	18.3 (0.3–70.9)
	
*Nilotinib therapybeforeTFR phase entry*	
Duration of nilotinib therapy, median (range) (months)	43.5 (32.9–88.7)
Actual nilotinib dose intensity during consolidation phase, median (range) (mg per day)	600 (400–600)

Abbreviations: CML, chronic myeloid leukemia; MR^4.5^, molecular response 4.5 (*BCR-ABL1*⩽0.0032% on the International Scale); TFR, treatment-free remission.

**Table 2:** Frequently reported AEs and laboratory abnormalities in the TFR population during the consolidation and TFR phases

*Patients,*n*(%)*	*Consolidation phase (*n*=190)*^a^		*TFR phase (*n*=190)*^a^	
	*Any grade*	*Grade 3/4*	*Any grade*	*Grade 3/4*
*AEs reported in >5% of patients in the consolidation or TFR phase*				
Nasopharyngitis	21 (11.1)	0	16 (8.4)	0
Arthralgia	16 (8.4)	0	23 (12.1)	2 (1.1)
Hypertension	15 (7.9)	10 (5.3)	7 (3.7)	2 (1.1)
Diarrhea	11 (5.8)	0	8 (4.2)	1 (0.5)
Headache	10 (5.3)	0	10 (5.3)	0
Pain in extremity	5 (2.6)	0	12 (6.3)	0
				
*Key hematologic abnormalities based on CTCAE grades of laboratory values*				
Anemia	46 (24.2)	0	38 (20.0)	1 (0.5)
Lymphopenia	17 (8.9)	1 (0.5)	26 (13.7)	0
Thrombocytopenia	16 (8.4)	0	20 (10.5)	0
Leukopenia	5 (2.6)	0	11 (5.8)	1 (0.5)
				
*Key biochemical abnormalities based on CTCAE grades of laboratory values*				
Elevated glucose	75 (39.5)	1 (0.5)	37 (19.5)	1 (0.5)
Elevated ALT	71 (37.4)	0	24 (12.6)	0
Elevated AST	30 (15.8)	0	13 (6.8)	0
Elevated bilirubin	57 (30.0)	3 (1.6)	6 (3.2)	0
Elevated lipase	57 (30.0)	6 (3.2)	22 (11.6)	3 (1.6)

Abbreviations: AE, adverse event; ALT, alanine aminotransferase; AST, aspartate aminotransferase; CTCAE, Common Terminology Criteria for Adverse Events; TFR, treatment-free remission.

a Median duration of study treatment during the consolidation phase among patients in the TFR population was 52 weeks, and the duration of the TFR phase was 48 weeks.

**Table 3:** AE groups of special interest^a^

*Patients,*n*(%)*	*Consolidation phase (*n*=190)*^b^	*TFR phase (*n*=190)*^b^
Musculoskeletal pain^c^	31 (16.3)	47 (24.7)
Fluid retention	4 (2.1)	8 (4.2)
Cardiovascular events	4 (2.1)	5 (2.6)
Ischemic cerebrovascular events	1 (0.5)	2 (1.1)
Ischemic heart disease	2 (1.1)	0
Peripheral artery disease	1 (0.5)	2 (1.1)
Others	0	1 (0.5)
Rash	8 (4.2)	2 (1.1)
Pancreatitis	3 (1.6)	0

Abbreviations: AE, adverse event; TFR, treatment-free remission.

a Each listed AE group includes a predefined set of individual AEs. Reported frequencies include all patients with ⩾1 AE in the group.

b Median duration of study treatment during the consolidation phase among patients in the TFR population was 52 weeks, and the duration of the TFR phase was 48 weeks.

c Defined as any of the following AEs: musculoskeletal pain, myalgia, pain in extremity, arthralgia, bone pain and/or spinal pain.

**Table 4:** Rate of musculoskeletal pain-related events reported during the TFR phase according to baseline characteristics

*Patients,*n/N*(%)*^a^	*Rate of musculoskeletal pain-related events*
*Age*^b^	
<55 years	23/94 (24.5)
⩾55 years	26/96 (27.1)
	
*Sex*	
Female	31/94 (33.0)
Male	18/96 (18.8)
	
*Time from achievement of MR*^*4.5*^ *until TFR phase entry*^b^	
<30.37 months	24/95 (25.3)
⩾30.37 months	25/95 (26.3)
	
*Duration of nilotinib before TFR phase entry*^b^	
<43.47 months	22/94 (23.4)
⩾43.47 months	27/96 (28.1)
	
*Musculoskeletal pain-related events before TFR phase entry*^c^	
Yes	15/49 (30.6)
No	34/141 (24.1)

Abbreviations: MR^4.5^, molecular response 4.5 (*BCR-ABL1*⩽0.0032% on the International Scale); TFR, treatment-free remission.

a Rates of musculoskeletal pain-related events reported during the TFR phase are shown as a percentage of patients within each subgroup.

b Subgroups were defined based on the median values for each parameter.

c Includes events in patient history before study entry or reported during the consolidation phase.
