# Supplementary material for: Treatment-free remission following frontline nilotinib in patients with chronic myeloid leukemia in chronic phase: results from the ENESTfreedom study
Source: Leukemia. 2017 Mar 17;31(7):1525–31. doi: 10.1038/leu.2017.63 (PMC5508077; doi:10.1038/leu.2017.63)
Supplement: Supplementary Information [file leu201763x1.pdf]

Supplementary Appendix

Supplement to Hochhaus A, Masszi T, Giles FJ, et al. Treatment-Free Remission Following Frontline Nilotinib in Patients With Chronic Myeloid Leukemia in Chronic Phase: Results From the ENESTfreedom Study

Supplementary Methods

Patients, study design, and treatment

During the 1-year continuation phase, molecular responses were evaluated every 12 weeks. Patients who entered the continuation phase were allowed to stop nilotinib treatment in the treatment-free remission (TFR) 2 (TFR-2) phase if they sustained a deep molecular response (DMR), defined as $MR^{4.5}$ (*BCR-ABL1* ≤ 0.0032% on the International Scale [*BCR-ABL1*^{IS}]) in the last assessment, no assessment worse than MR^{4} (*BCR-ABL1*^{IS} ≤ 0.01%), and ≤ 2 assessments between MR^{4} and $MR^{4.5}$, during the continuation phase. Patients in the continuation phase who are ineligible to enter the TFR-2 phase will remain on nilotinib treatment until 192 weeks after the last patient entered the TFR phase or until discontinuation due to intolerable toxicity, disease progression, investigator discretion, or withdrawal of consent.

Figure S1. Kaplan-Meier estimate of treatment-free survival[a] among patients who remained in the TFR phase for ≥ 24 weeks.

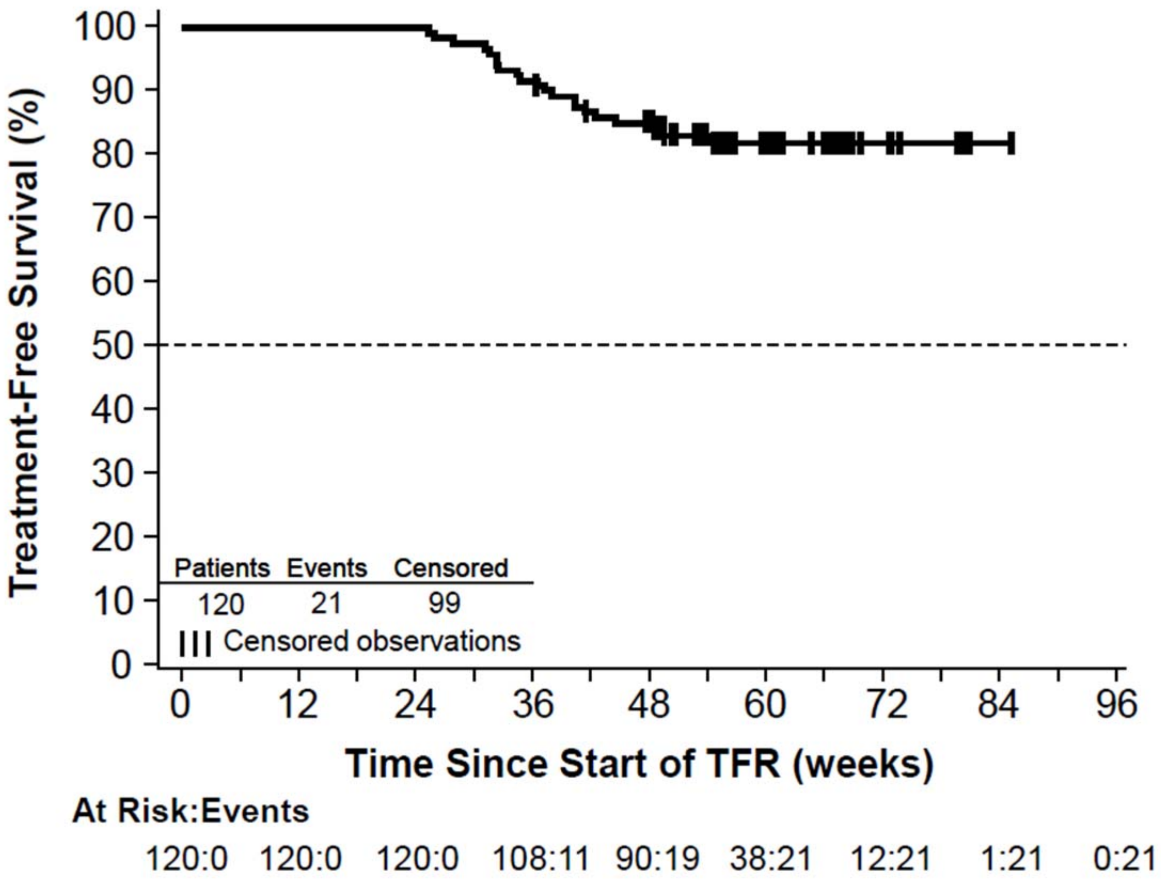

TFR, treatment-free remission.

[a] Treatment-free survival was defined as the time from the start of TFR until the earliest of any of the following: loss of major molecular response (*BCR-ABL1* ≤ 0.1% on the International Scale), reinitiation of nilotinib for any reason, progression to accelerated phase/blast crisis, or death due to any cause.

Table S1. Baseline characteristics of patients according to TFR status at 48 weeks

	**Patients Remaining in TFR at 48 Weeks (n = 98)**	**Patients No Longer in TFR at 48 Weeks (n = 92)**
Age, median (range), years	53.0 (21-83)	56.5 (23-86)
Sex, %		
Female	53.1	45.7
Male	46.9	54.3
Time from achievement of $MR^{4.5}$ to study entry, median (range), months[a]	19.3 (0.4-70.9)[b]	16.9 (0.3-33.4)[b]
Duration of nilotinib prior to study entry, median (range), months[a]	31.7 (21.2-76.6)[c]	31.2 (24.0-47.9)[c]

$MR^{4.5}$, molecular response 4.5 (*BCR-ABL1* ≤ 0.0032% on the International Scale); TFR, treatment-free remission.

[a] Per protocol, there was a fixed duration of 52 weeks between study entry and entry into the TFR phase. Thus, the total durations of $MR^{4.5}$ and nilotinib treatment increased similarly for all patients between the time of study entry and entry into the TFR phase.

[b] The interquartile range for time from achievement of $MR^{4.5}$ to study entry was similar in patients remaining in TFR (4.2-26.0 months) and in those no longer in TFR (1.5-21.3 months) at 48 weeks.

[c] The interquartile range for duration of nilotinib prior to study entry was similar in patients remaining in TFR (27.8-35.0 months) and in those no longer in TFR (27.1-33.9 months) at 48 weeks.

Table S2. TFR rate according to baseline characteristics

**Patients, n/N (%)**[a]	**TFR Rate at 48 Weeks**
Age[b]	
< 55 years	51/94 (54.3)
≥ 55 years	47/96 (49.0)
Sex	
Female	52/94 (55.3)
Male	46/96 (47.9)
Time from achievement of $MR^{4.5}$ to study entry[b,c]	
< 18.3 months	45/95 (47.4)
≥ 18.3 months	53/95 (55.8)
Duration of nilotinib prior to study entry[b,c]	
< 31.4 months	47/94 (50.0)
≥ 31.4 months	51/96 (53.1)

$MR^{4.5}$, molecular response 4.5 (*BCR-ABL1* ≤ 0.0032% on the International Scale); TFR, treatment-free remission.

[a] Rates of patients remaining in TFR at 48 weeks are shown as a percentage of patients within each subgroup.

[b] Subgroups were defined based on the median values for each parameter.

[c] Per protocol, there was a fixed duration of 52 weeks between study entry and entry into the TFR phase. Thus, the total durations of $MR^{4.5}$ and nilotinib treatment increased similarly for all

patients between the time of study entry and entry into the TFR phase; therefore, this 52-week period did not impact this analysis.
